# Synthesis, characterization and thermal behavior of plasticized poly (vinyl chloride) doped with folic acid-modified titanium dioxide

**DOI:** 10.1038/s41598-022-07177-5

**Published:** 2022-03-01

**Authors:** Yi-heng Lu, Zong-lin Chen, Yu-wei Lu

**Affiliations:** 1 School of Chemical Engineering, Anhui University of Science and Technology, Huainan, 232001 China; 2 Laboratoire de Chimie Physique, Universite de Paris Sud, 91405 Orsay Cedex, France

**Keywords:** Environmental sciences, Chemistry, Engineering, Materials science

## Abstract

To inhibit the agglomeration of nanotitanium dioxide, a poly (vinyl chloride) (PVC) composite film doped with folic acid-modified titanium dioxide was synthesized and characterized using X-ray powder diffraction, X-ray photoelectron spectroscopy and thermogravimetric analysis coupled with Fourier transform infrared spectroscopy. The average grain size of the folic acid-modified titanium dioxide was found to decrease by 1.3 nm, indicating that the cohesiveness of the nanoparticles is decreased. The lowest temperature for 1.0% thermal decomposition of PVC was determined to be 230.0 °C. The decomposition rate at the peak temperature is found to be 39.6% lower than that of a control sample. The stability of the PVC is improved due to a lower number of surface chlorine atoms as well intermolecular attraction. A mechanism for folic acid modification of titanium dioxide-doped PVC is proposed. After doping, the ester groups in the plasticizer show a significant decrease in the vibration peak intensities observed at 1264 cm^−1^, 1736 cm^−1^ and 1106 cm^−1^. The doped PVC film suppresses the release of CO_2_, and the strongest vibration peak at 1264 cm^−1^ is found to be 17.2% lower than that for the blank sample, indicating that doping is beneficial for plasticizer recovery.

## Introduction

As a thermoplastic polymer material, poly (vinyl chloride) (PVC) is widely used in building materials, packaging, electrical cables, and medical applications because of its low price, ease of processing, corrosion resistance, and flame resistance. However, PVC has the disadvantages of poor thermal stability and easy release of corrosive hydrogen chloride gas during molding and processing, resulting in performance degradation^1^. Commonly used stabilizers, costabilizers and auxiliary agents include hydrogen chloride traps or barriers that cut off the transmission of chlorine free radicals, such as calcium/zinc metal soaps, organotin, organic compounds (such as polyhydroxy compounds), rare earths, nanometal oxide particles and lead salts^2–6^. With increasing pressure to protect the environment, there is an urgent need for new composite stabilizers that are efficient, nontoxic, environmentally degradable or recyclable.

PVC/TiO_2_ organic/inorganic nanocomposites are new materials that have attracted attention in recent decades. These materials combine inorganic nanoparticles in a polymer matrix, and, in general, show greatly improved mechanical and optical properties^7–11^. They are nontoxic, stable and resistant to ultraviolet radiation^12–14^. For example, titanium conversion coatings on aluminum foil AA 8021 are used for lithium–ion battery packaging^15^. The preparation and application of PVC for novel organic/inorganic composite UV absorbers has been carried out^16^. The photostabilization efficiency of ultraviolet light stabilizers for rigid polyvinyl chloride) against photooxidation has been investigated^17^. The kinetics of poly (vinyl chloride)/titanium dioxide nanocomposites photodegradation under accelerated ultraviolet and visible light exposure has been studied^18^. Preparation of the cross-linked chitosan-coated calcium sulfate whisker and its reinforcement in PVC has been investigated^19^. Nano TiO_2_ (NT) particles can effectively degrade a variety of pollutants and avoid the formation of highly toxic substances, such as dioxins^20–24^. However, NT particles have a large specific surface area and high surface energy, so they readily agglomerate and are difficult to disperse in a polymer. NT particles are inorganic metal oxide particles with strong polarity and a large number of hydroxyl groups on their surface, which are incompatible with PVC polymers because they are repelled. Therefore, there is an urgent need to solve the high surface energy, high hydrophilicity and repellency of NT with PVC.

Folic acid (FA) contains polar and nonpolar groups and is one of the candidate ligands. It is a pure natural vitamin that is safe and nontoxic. It contains multiple amino and carboxyl groups and shows weak acidity. As a ligand, it can form complexes with metals. The NT surface contains OH, which can combine with the carboxyl and amino groups on the FA macromolecular structure to produce an electrostatic attraction, remove hydroxyl groups from the NT surface, reduce hydrophilicity and improve hydrophobicity. In this way, the compatibility and affinity of FA/NT with PVC can be increased.

To date, there are many reports of PVC composites that contain titanium dioxide, show photocatalytic antibacterial and mechanical properties and are involved in photocatalytic degradation^25–36^, but the heat resistance of PVC composites has not been improved. Therefore, the heat resistance of modified NT-reinforced PVC composites is a long-term issue that needs to be solved. In this work, an FA surface modification NT complex-reinforced PVC composite film was synthesized to improve the dispersion of NT, reduce cohesion, increase compatibility with the PVC substrate and increase the environmental friendliness. The hydrogen chloride release rate of the film during thermal degradation was measured, the films were characterized by X-ray powder diffraction (XRD), Fourier transform infrared spectroscopy (FTIR) and X-ray photoelectron spectroscopy (XPS), and the effect of FA/NT on the thermal stability and stability mechanism of the films was investigated. This study has never been reported in the literature. In addition, possible synergistic effects and the thermal behavior of FA/NT, CeSt_3_ and Organotin (OT) composites used on PVC substrates were also investigated. This study is beneficial to the development of a new type of environmentally friendly PVC/FA/NT composite film.

## Results

### Removal of hydrogen chloride by thermal decomposition of different films

**Scheme 1:**
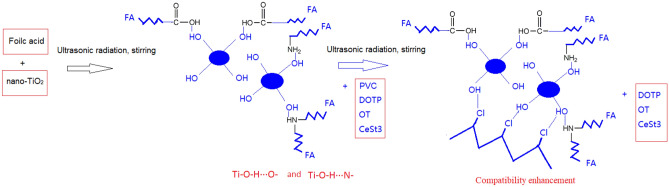
Formation mechanism for the PVC/FA/NT composites.

**Scheme 2:**
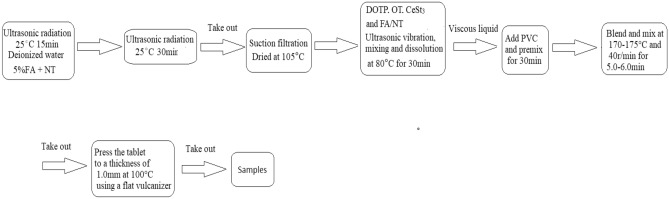
Schematic diagram of the procedure used for the preparation of FA/NT/PVC composites.

The mechanism and procedure for the preparation of PVC/FA/NT composites are schematically shown in Schemes 1 and 2.

As can be observed from Scheme 1, a relatively stable attractive force exists between PVC and FA/NT. After doping, the compatibility between PVC and FA/NT is significantly enhanced due to the increase in hydroxyl groups and the formation of stable hydrogen bonds with Cl atoms.

Scheme 2 shows a schematic diagram of the procedure used for the preparation of PVC/FA/NT composites, including the synthesis of FA/NT and the setting of ultrasonic reaction temperature, time and ratio. The preparation of FA/NT-doped PVC composite film materials was performed through DOTP, OT, cerium stearate and FA/NT stirring and dissolution, dispersion in PVC resin, twin-screw extrusion, internal mixing, mixing and other process steps.

**Scheme 3:**
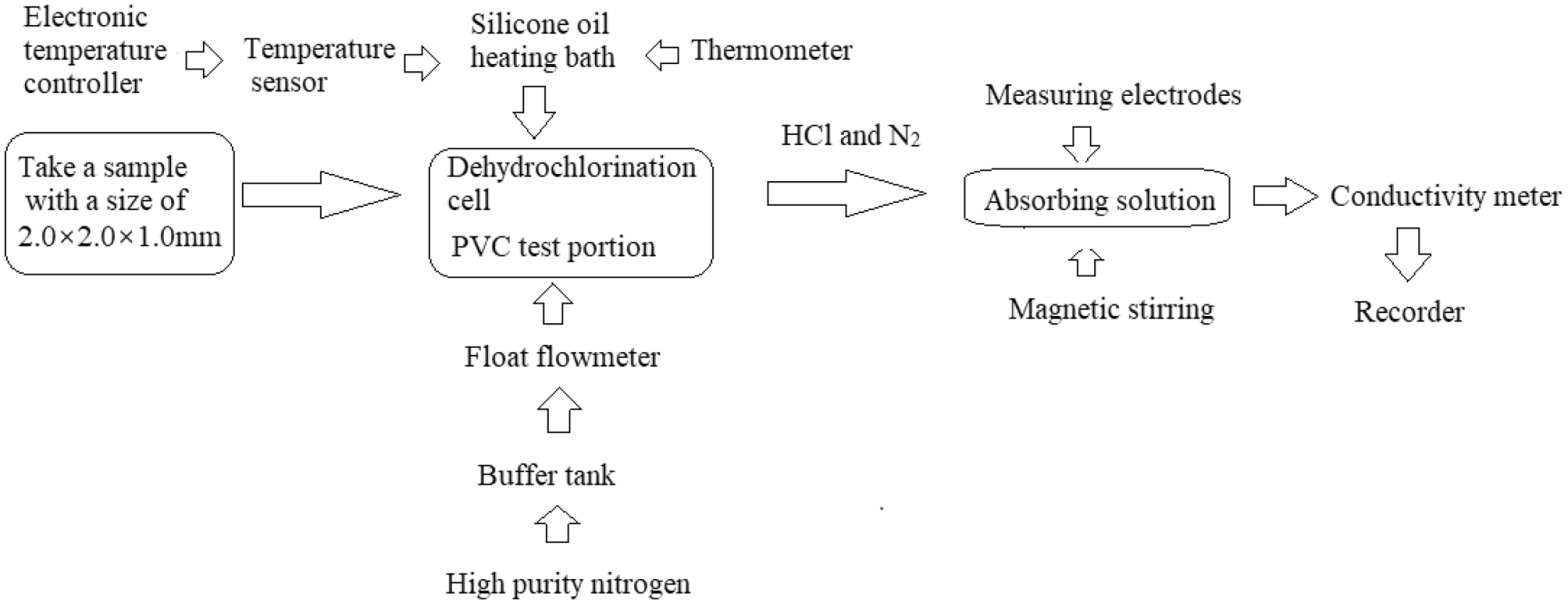
Schematic diagram of the procedure used for determination of the conductivity of the HCl-released aqueous solution.

Scheme 3 shows a schematic diagram of the procedure used for the determination of the conductivity of the HCl released as aqueous solution. The conductivity was measured in accordance with the ENIS0182-3:2000 standard.

**Figure 1:**
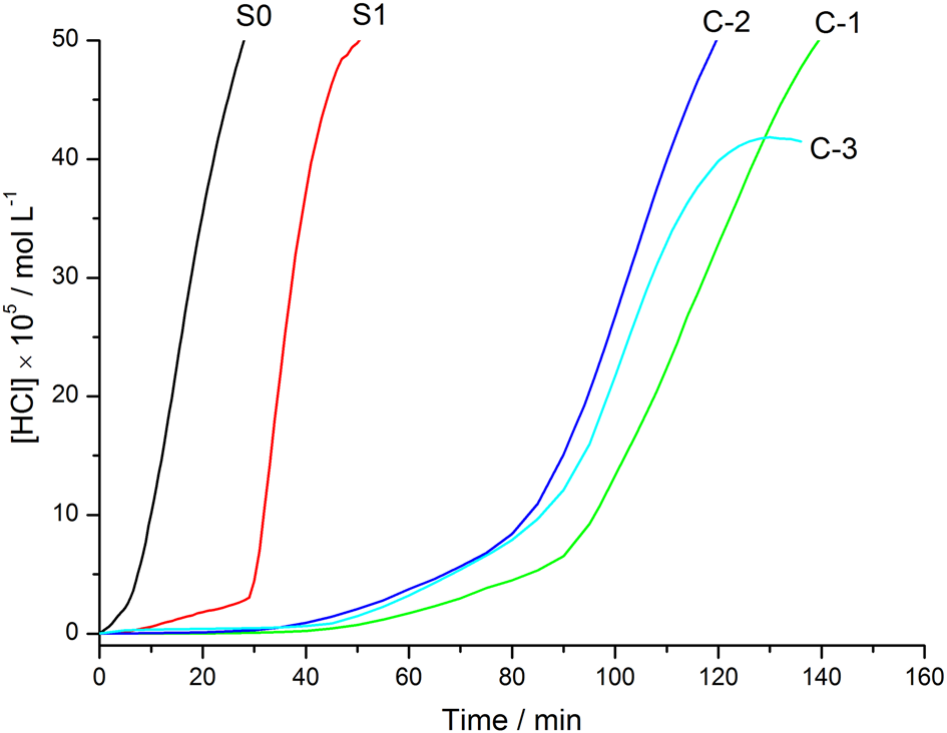
The change in the concentration of aqueous hydrogen chloride solution released by different PVC films over time (at 195 °C and under a N_2_ atmosphere).

Figure 1 shows the change in the concentration of aqueous hydrogen chloride solution released by thermal degradation of different PVC films over time. In this figure, the abscissa is time (min) and the ordinate is hydrogen chloride concentration ([HCl] × 105 mol L^−1^). The concentration of aqueous hydrogen chloride solution at different times can be obtained by conductivity conversion according to Eq. (1)^37^. The conductivity of the aqueous hydrogen chloride solution measured at different times was obtained according to the ENIS0182-3:2000 standard. The stability of the PVC film S1 with 1 phr OT is higher than that of S0 without any additives.

The thermal degradation of the PVC/FA/NT film and release of hydrogen chloride are obviously inhibited, and the thermal stability of C-1 is the best. At 195 °C and with high purity nitrogen, the order of hydrogen chloride release is S0 > S1 > C-2 > C-3 > C-1. This indicates that S0 is the most unstable, followed by S1 and C-2, which are close to C-3, and C-1 peaks last. It can be observed that the film stability from strong to weak order is C-1 > C-3 > C-2 > S1 > S0.

Table 1 shows the induction period for different PVC films that release hydrogen chloride during thermal degradation. τ_i_ represents the induction period (min), τ_m_ represents the maximum value (min), and τi is the intersection of the bottom horizontal line and the vertical tangent for hydrogen chloride shown in Fig. 1. The induction period of C-1 is 70 min, which is the longest, and the induction periods of C-3, C-2, S1 and S0 are 59 min, 56 min, 28 min and 5 min, respectively. This trend shows that FA/NT, CeSt_3_ and OT strongly inhibit the release of hydrogen chloride from the PVC film or strongly interact with hydrogen chloride. The test results show that with the introduction of 1–3 phr FA/NT and 5 phr CeSt_3_, the stabilizing effect is far greater than that with 1.0 phr OT. OT enhances the ability of the other chemical agents to react with hydrogen chloride in the PVC. When the OT is partially replaced, the FA/NT and CeSt_3_ complexes show a better synergistic effect on the OT; this effect is especially obvious when 1 phr FA/NT is added.

**Table 1:** Induction period for different PVCs that release hydrogen chloride.

Sample	τ_i_/min	τ_m_/min
S0	5	27
S1	28	49
C-1	70	138
C-2	56	119
C-3	59	128

*τ*_*i*_ induction period; *τ*_*m*_ maximum value, that is [HCl] reaches 50 × 10^–5^ mol L^−1^, C-3 is 41 mol L^−1^).

### Fourier transform reflectance infrared spectroscopy analysis of different films

**Figure 2:**
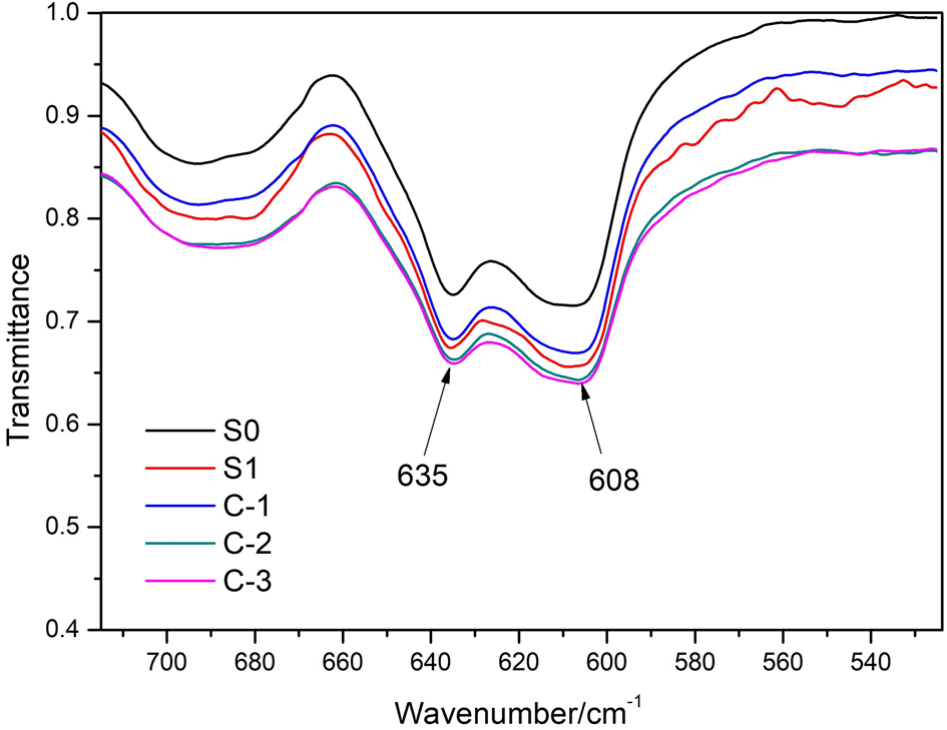
FTIR reflectance spectra for the different PVC films.

Figure 2 shows the FT-IR spectra for different PVC films in the wavenumber range from 525–715 cm^−1^, in which the vibration peak near 608 cm^−1^ is attributed to characteristic groups of the C–Cl bond^38–40^. The width of the vibration peak from the C–Cl bond on the surface of the PVC film after FA/NT modification is slightly larger than that of the control and blank samples. The peak widths from large to small follow the order of C-3 > C-2 > S1 > C-1 > S0, where the peak widths for C-3, C-2 and S1 are 7 cm^−1^ larger than those for C-1 and S0. The differences in the vibration peaks in the wavenumber range from 715–4000 cm^−1^ before and after the modification are small. The reason for this may be that the surface coating and content of FA/NT only ranges from 0.64 to 3.12%.

### X-ray diffraction analysis of different films

**Figure 3:**
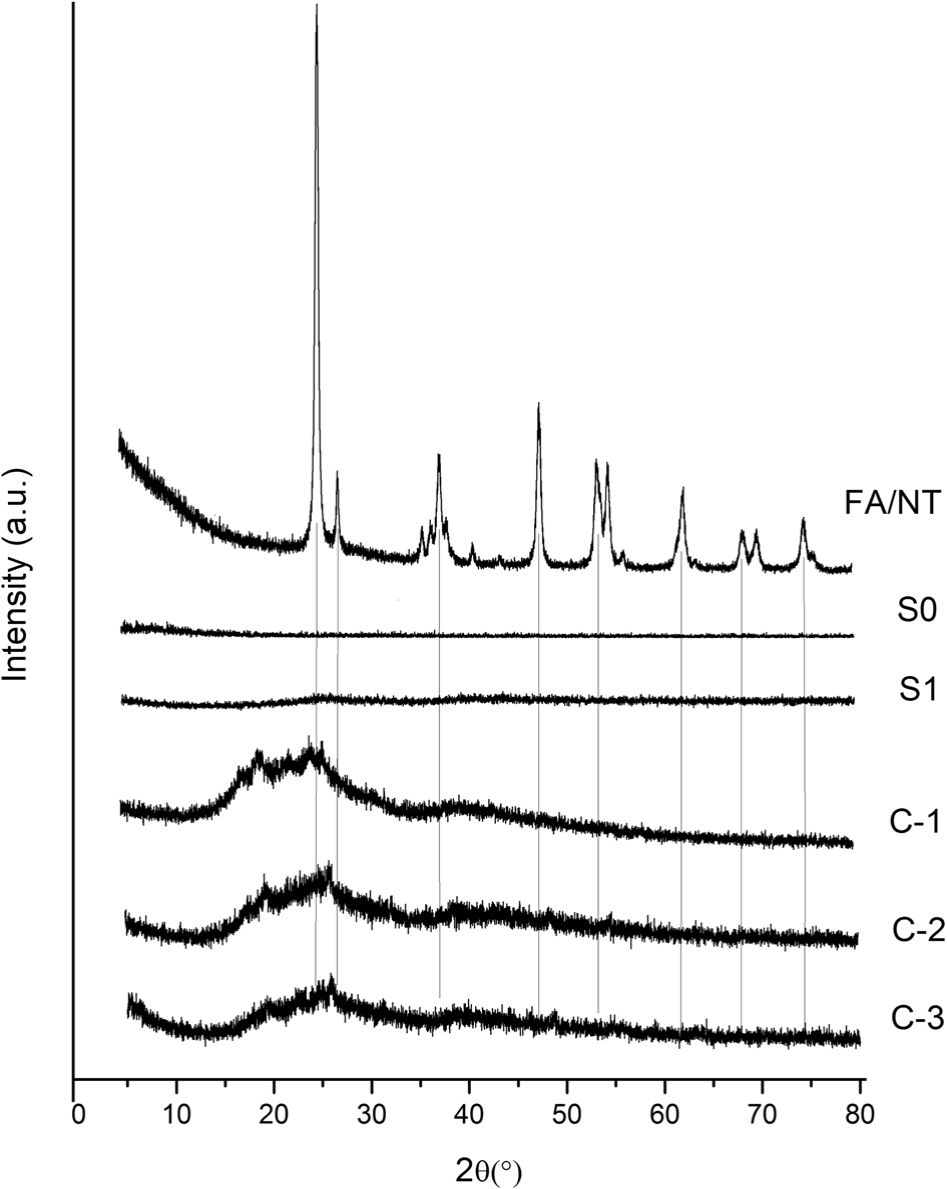
XRD profiles for different PVC films.

Figure 3 shows the XRD profiles for different PVC films. It can be observed from this figure that S0 and S1 show almost no characteristic diffraction peaks, and no crystalline particles exist in the samples. The characteristic peaks for FA/NT observed at 25.3°, 37.7°, 48.0°, 53.9° and 55.1° are attributed to the (101), (004), (200), (105) and (211) NT diffraction peaks, respectively. After doping with FA/NT, C-1, C-2 and C-3 generate diffraction peaks in the 2θ range from 15.0° to 75.1°, and the diffraction peaks between 25.3° are the most significant. This indicates that FA/NT and PVC show a strong interaction, and they are located on the PVC surface^6^. The diffraction peak observed for 2θ ranging from 15.0° to 30.0° is inferred to be the result of the interaction between FA/NT and PVC. The diffraction peak intensity from large to small follows the order of C-1 > C-2 > C-3, indicating that the crystallinity of the sample decreases with increasing FA/NT complex content. This trend is similar to the trend observed in the hydrogen chloride release experiment. As the content of FA/NT is increased, the stability of PVC during thermal degradation is decreased.

### X-ray photoelectron spectroscopic analysis of different films

**Figure 4:**
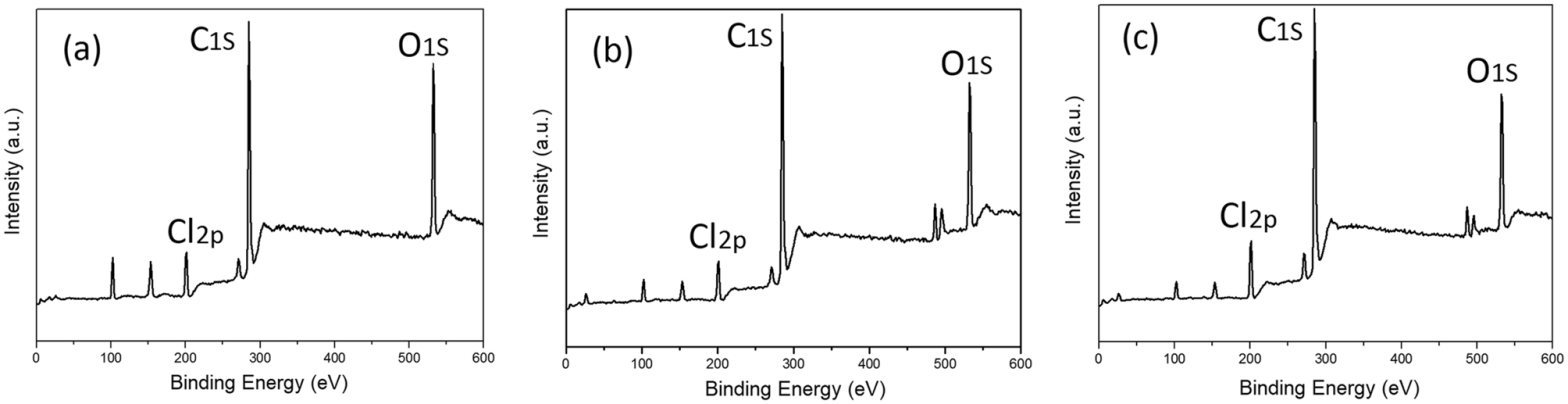
XPS spectra for PVC films (**a**) C-1, (**b**) C-2 and (**c**) C-3.

XPS spectra for the C-1, C-2 and C-3 films are shown in Fig. 4. Unlike the C-1 film, new Sn peaks are observed for C-2 and C-3 at 486.3 eV and 495.7 eV, respectively.

**Figure 5:**
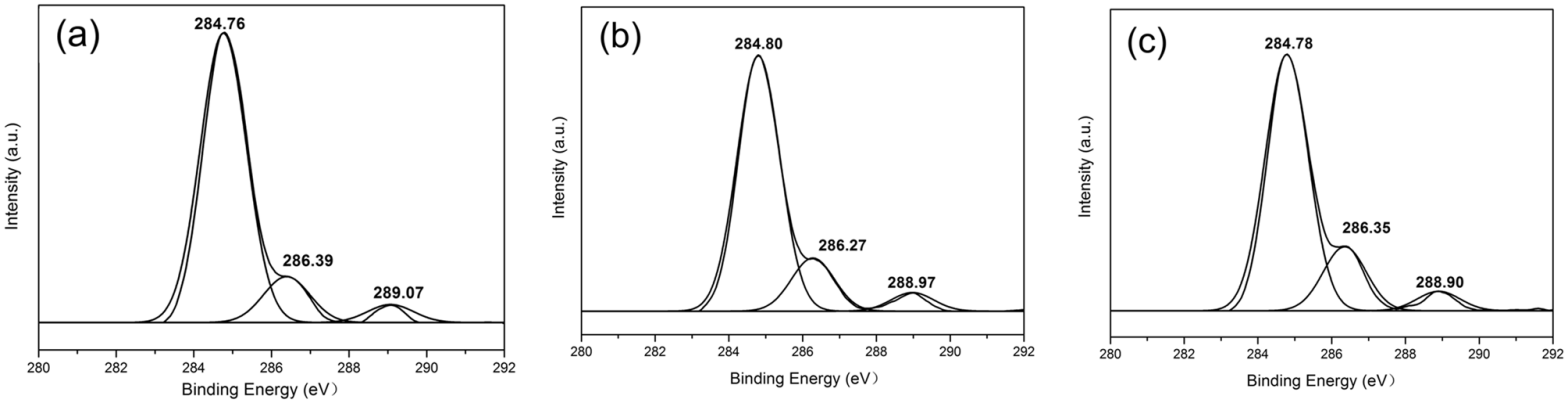
XPS spectra obtained from high-resolution scans of the C 1s region of (**a**) C-1, (**b**) C-2 and (**c**) C-3.

The high-resolution spectra for the C 1s regions are depicted in Fig. 5a–c. From the C 1s spectrum of the C-1, C-2 and C-3 films, the three components 1, 2 and 3 in the C ls spectra correspond exactly to three types of carbon bonds: C–C (284.76 eV), C–O and C–N (286.39 eV) and amide C=O (289.07 eV).

**Figure 6:**
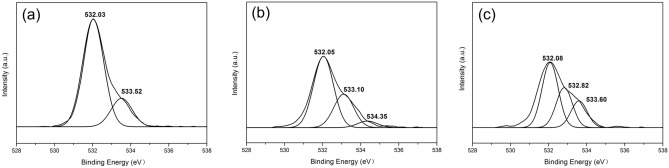
XPS spectra obtained from high-resolution scans of the O 1s region of (**a**) C-1, (**b**) C-2 and (**c**) C-3.

As displayed in Fig. 6a, two different types of oxygen components, O=C (532.03 eV) and O–C (533.52 eV), are found to be present in the O 1s spectrum of the C-1 film. From the O 1s spectrum obtained from high-resolution scans of the O 1s region for C-2 and C-3 shown in Fig. 6b,c, although oxygen groups (O=C) still remain, the peak intensities for the C-1 groups (O=C) show an obvious decrease. With increasing amount of added FA/NT, a new peak is generated near 534.35 eV, indicating that the carbonyl group is involved, and the carbonyl group is derived from DOTP and FA.

**Figure 7:**
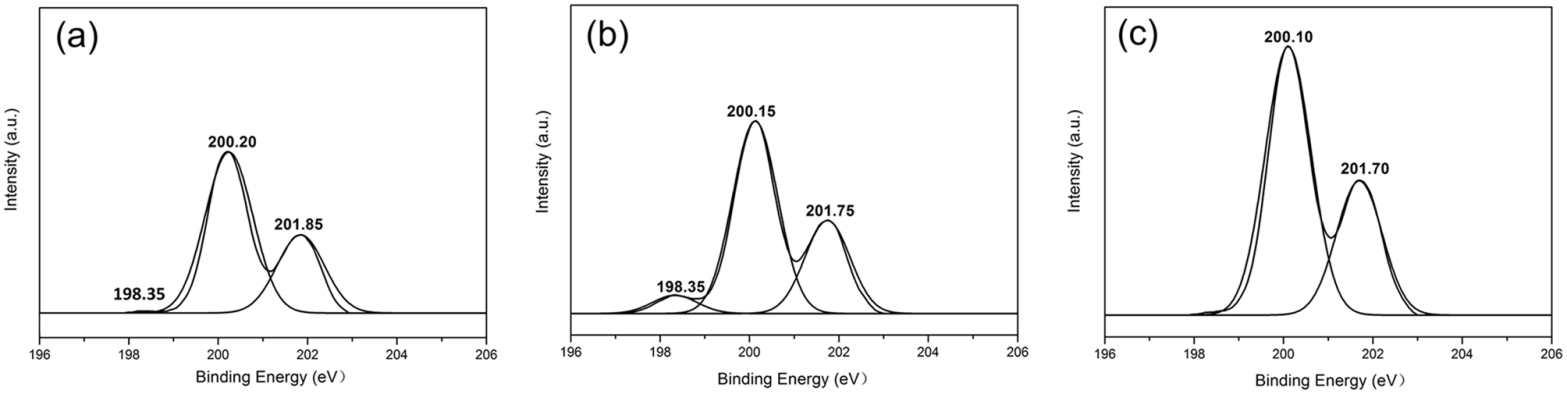
XPS spectra obtained from high-resolution scans of the Cl 2p region of (**a**) C-1, (**b**) C-2 and (**c**) C-3.

As depicted in Fig. 7c, the high-resolution Cl 2p spectrum of the C-3 film shows that two different chlorine components exist at 200.10 eV and 201.70 eV. In contrast, in the spectra shown in Fig. 7a,b, new peaks centered at 198.35 eV and 198.35 eV appear that are assigned to hydrogen bonds. The former peak corresponds to a weaker C-1 hydrogen bond.

The XPS binding energy data for the composite films are shown in Table 2; see attachment for the XPS spectra for S0 and S1. There are many possible sources of C 1s in the sample, including the increase in FA content in the FA/NT, resulting in an increase in C 1s content.

**Table 2:** XPS element distributions.

Sample	C 1s (%)	O 1s (%)	Cl2p (%)	O/C	Cl/C
C-1	76.14	19.38	4.48	0.25	0.06
C-2	78.10	15.96	5.94	0.20	0.08
C-3	77.37	14.93	7.70	0.19	0.10
S0	76.18	17.01	6.81	0.22	0.09
S1	74.46	17.80	7.74	0.24	0.10

See attachment for the XPS spectra for S0 and S1.

The content of surface O 1s in C-1, C-2 and C-3 is decreased in turn, and the O/C ratio drops from 0.25 to 0.19. The order of the oxygen carbon ratio from large to small is C-1 > S1 > S0 > C-2 > C-3. The main O 1s contribution arises from DOTP, followed by contributions from OT and FA. This is due to O atoms, such as those in FA and DOTP, involved in hydrogen bond formation in the PVC system, resulting in an increase in the surface oxygen atom concentration.

The content of surface Cl 2p in C-1, C-2 and C-3 increases in turn, and the Cl/C ratio is increased from 0.06 to 0.10. The chlorine carbon ratios for C-3, S0 and S1 are similar, indicating that the chlorine content on the surface shows little change. This shows that in C-3, there is no force between the C–Cl bond and FA/NT in PVC. The Cl2p signals mainly arise from the PVC resin. This result indicates that with increasing FA/NT, the amount of surface chlorine that is not involved in the formation of hydrogen bonds on the C-2 and C-3 films increases.

Therefore, the 1 phr FA/NT complex shows that a strong interaction exists between the hydroxyl group in NT and the C–Cl bond between PVC molecules, resulting in a low chlorine content on the surface of the composite, which leads to an improvement of the heat resistance.

### Thermogravimetric analysis

**Figure 8:**
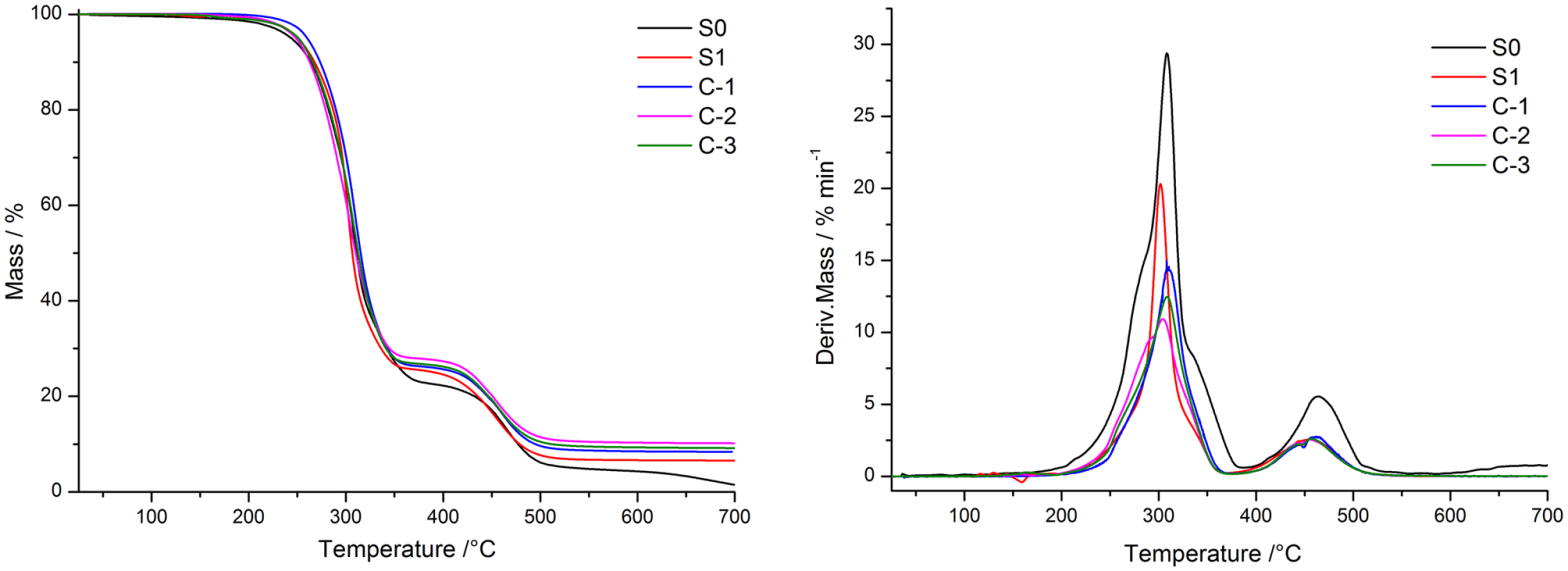
TG-DTG curves for different PVC films.

Figure 8 shows the TG-DTG curves for the different PVC films. The carrier gas was N2, and the heating rate was 10 °C min^−1^. The figure shows that the thermal degradation of the different PVC films can be separated into two stages, with the first and second stages of degradation occurring in the temperature ranges of 201–385 °C and 385–547 °C, respectively. During each decomposition stage, C-1 is better than S1 and the other samples, as it withstands higher temperatures. During the first decomposition stage, when the conversion rate is 1%, the initial decomposition temperature of C-1 is 230.0 °C, while the initial decomposition temperatures of S0 and S1 are 175.1 °C and 210.8 °C, respectively. The temperature for C-1 is significantly higher than that of S0 and S1.

Table 3 shows the decomposition temperatures of the PVC films for different conversion rates. When the conversion rate is 5%, the thermal degradation temperatures of C-1, C-2, C-3, S1 and S0 are 260.0 °C, 251.3 °C, 250.5 °C, 248.6 °C and 244.1 °C, respectively, showing a decreasing trend. C-1 degrades at the highest temperature of 260.0 °C, and the shift to the high-temperature zone is the most obvious.

**Table 3:** The decomposition temperatures of the PVC films at different conversion rates.

α/%	S0	S1	C-1	C-2	C-3
	/°C				
1	175.1	210.8	230.0	212.7	198.2
5	244.1	250.5	260.0	248.6	251.3
10	264.1	267.7	272.9	262.7	265.8
80	432.1	435.7	447.7	451.0	446.6
90	477.1	478.6	495.0	703.5	511.4

Table 4 shows the decomposition rates of the PVC films at different temperatures. The decomposition rates of C-1, C-2, C-3, S1 and S0 during the first decomposition stage at 300 °C were determined to be 12.55% min^−1^, 10.53% min^−1^, 10.85% min^−1^, 19.81% min^−1^ and 22.75% min^−1^, respectively. The decomposition rate of PVC after modification decreased significantly compared with that of the control and blank samples.

**Table 4:** The decomposition rates for the PVC film at different temperatures.

T/°C	S0	S1	C-1	C-2	C-3
	% min^−1^				
200	0.619	0.168	0.120	0.209	0.155
300	22.75	19.81	10.85	10.53	10.85
400	0.736	0.603	0.400	0.379	0.384
450	4.334	2.513	2.210	2.384	2.289
500	1.714	0.602	0.630	0.585	0.586
700	0.797	0.008	0.006	0.022	0.022

Table 5 shows the characteristic temperatures of the different PVC films. The table shows that during the first decomposition stage, C-1, C-2, C-3, S1 and S0 show peak temperatures of 309.5 °C, 304.2 °C, 308.5 °C, 302.2 °C and 308.1 °C, respectively, and the corresponding decomposition rates are 14.51% min^−1^, 10.91% min^−1^, 12.47% min^−1^, 20.26% min^−1^ and 29.40% min^−1^, respectively. The peak temperature for C-1 is 309.5 °C, which is obviously in the high-temperature zone and higher than that for S1 and S0. The decomposition rate of C-1 at the peak temperature is 39.6% and 102.6% lower than that of S1 and S0, respectively. During the second decomposition stage, the maximum decomposition rate of S0 is 5.55%/min, and the rest of the rates are similar and decreased to 2.5–2.7%/min.

**Table 5:** Characteristic temperatures of different PVC films.

Sample	S0	S1	C-1	C-2	C-3	Stage
T_p_/°C	308.1	302.2	309.5	304.2	308.5	1st
dα/dt (%min^−1^)	29.40	20.26	14.51	10.91	12.47	
T_p_/°C	464.1	455.8	464.8	454.5	456.1	2nd
dα/dt (%min^−1^)	5.55	2.57	2.67	2.59	2.61	

### TG-FTIR analysis

**Figure 9:**
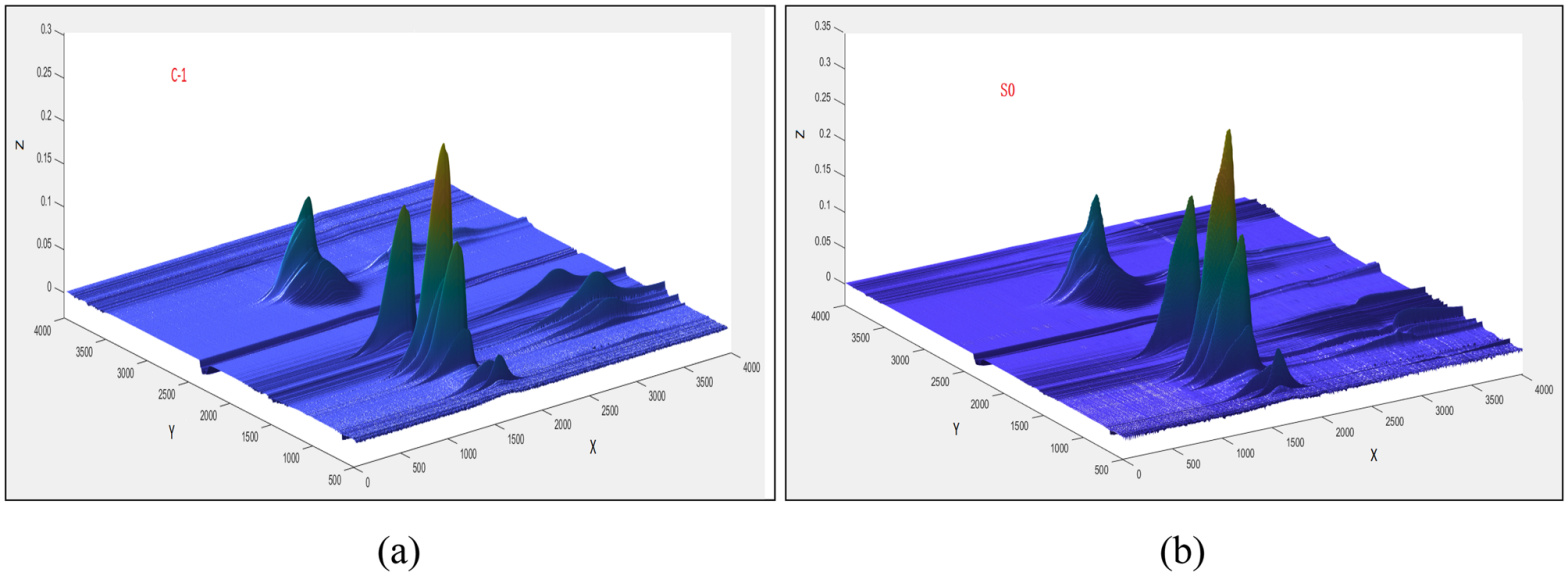
3D TG-FTIR spectrum of the gas phase in the thermal degradation of (**a**) C-1 and (**b**) S0 (time: 0–4000 s; temperature range: room temperature—700 °C; wavenumber: 500–4000 cm^−1^, β = 10 °C/min, carrier gas: high purity nitrogen; X-time/s; Y-wavenumbers/cm^−1^; Z-Absorbance).

The gas phase 3D TG-FTIR spectra measured for the C-1 and S0 sample pyrolysis are shown in Fig. 9, where the x coordinate is time/second, the y coordinate is wavenumbers/cm^−1^, and z is the absorbance. Figure 6a shows a TG-IR perspective for the C-1 sample. There are four stronger infrared absorption peaks at 1621 s, 1626 s, 1674 s and 1653 s, with absorbance peak intensities of 0.2562, 0.1604, 0.1498 and 0.1085, respectively.

**Figure 10:**
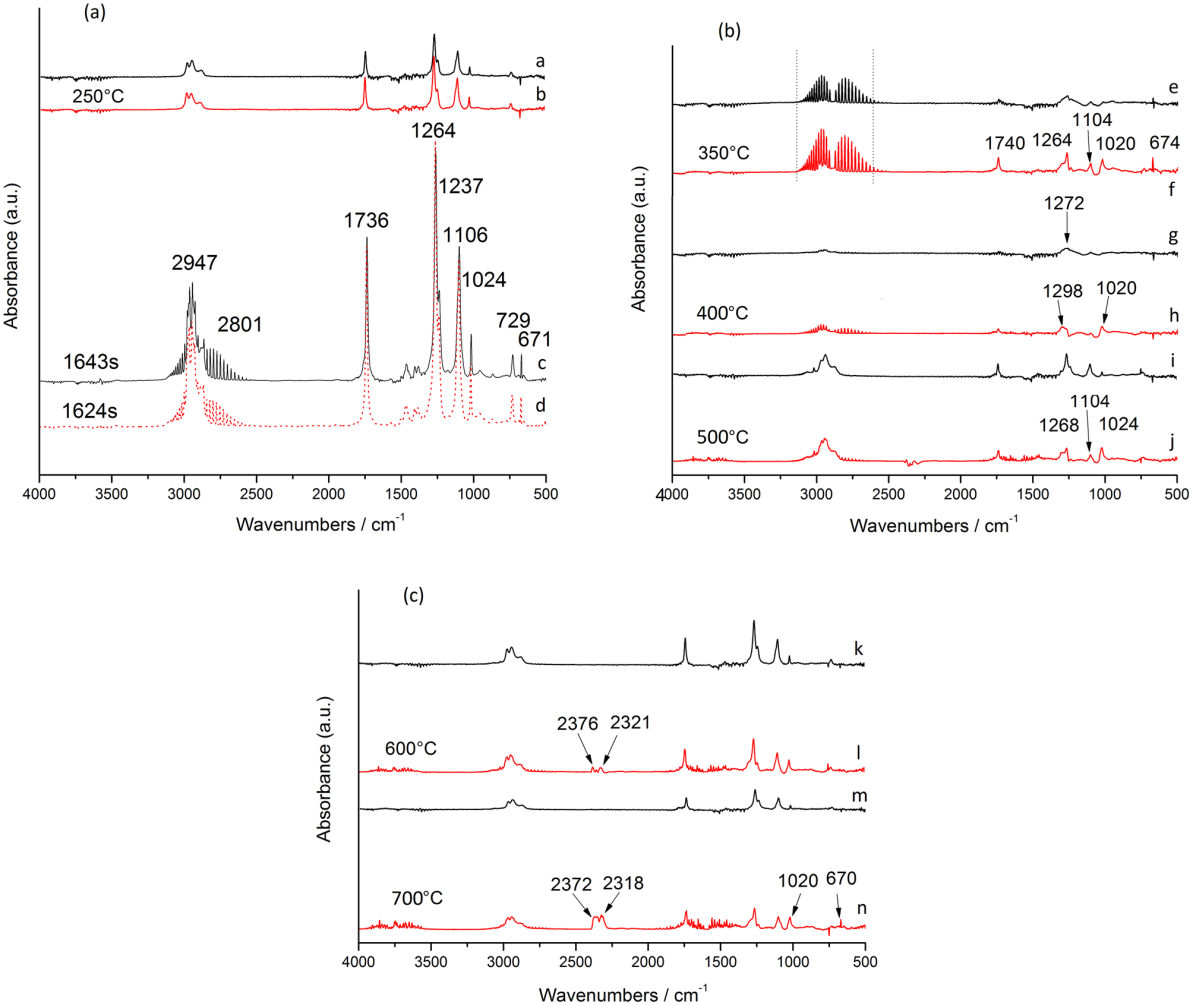
FTIR spectra measured for PVC at different temperatures (black is C-1; red is S0; (**a**,**b**) 250 °C; (**c**) 1643 s (time), (**d**) 1624 s (time); (**e**,**f**) 350 °C; (**g**,**h**) 400 °C; (**i**,**j**) 500 °C; (**k**,**l**) 600 °C; (**m**,**n**) 700 °C).

Figure 10 shows the infrared spectra for the released gas at different temperatures, wherein the black curve represents C-1 and the red curve represents S0. As shown in Fig. 10a, a and b show the infrared spectra for the gas phase at 250 °C. A small amount of gas is released, but the difference is not significant. c and d show the vibration peaks at pyrolysis times of 1643 s (310.8 °C) and 1624 s (307.7 °C), respectively, which include the hydrogen chloride removal stage where C-1 is shifted to the high-temperature zone.

The vibration peak near 1264 cm^−1^ is attributed to esters from DOTP in C-1. Its intensity is significantly lower than that of S0, and the peak intensity decreases by 17.3%. The vibration peaks near 2950 cm^−1^ and 2798 cm^−1^ are attributed to hydrogen chloride gas^41^, the peaks near 1736 cm^−1^, 1264 cm^−1^, 1106 cm^−1^ and 1024 cm^−1^ are attributed to the ester group ν_(COOR)_ from the plasticizer DOTP^42^, and the 671 cm^−1^ peak is attributed to the stretching of the ν_(C–Cl)_ groups^41,43^. The peak strength of C-1 after modification obviously decreases compared with S0, which shows that the C-1 heat resistance is enhanced.

The absorption peak shown in Fig. 10b shows that upon heating from 350 to 500 °C, the vibration peaks at 2600–3100 cm^−1^ disappear first, and then a weaker hydrocarbon vibration peak is generated. The vibration peaks near 1736 cm^−1^, 1264 cm^−1^, 1106 cm^−1^, 1024 cm^−1^ and 671 cm^−1^ almost disappear and become weaker, indicating that DOTP gasification products are minimal and that the removal of hydrogen chloride is almost complete. The conjugated polyenes start to polymerize. Here, the infrared spectra e, g and i represent C-1; f, h and j represent S0.

As shown in Fig. 10c, the second stage of PVC thermal degradation occurs at 600 °C, and the vibration peak of carbon dioxide is observed between 2318 and 2372 cm^−1^. During this stage, S0 begins to release CO_2_, while C-1 inhibits CO_2_ overflow. At 700 °C, the peak height for the signal from the CO_2_ released from S0 is further increased compared with that from C-1, which also inhibits CO_2_ release and inhibits the degradation of the plasticizer DOTP, facilitating the recovery of DOTP. In the infrared spectra, k and m represent C-1, and l and n represent S0. Therefore, after enhancement, the intensities of the vibration peaks at 1264 cm^−1^, 1736 cm^−1^, 1106 cm^−1^ and 1024 cm^−1^ were due to a significant decrease in DOTP, which shows that the additive has a strong interaction with DOTP, which inhibits the thermal degradation of PVC and improves the thermal stability of the C-1 film.

### Stability mechanism of the FA/NT/PVC composites

As can be observed from Scheme 1, first, FA contains not only polar groups, such as carboxyl and amide groups but also nonpolar groups, such as carbon chain skeletons. After the FA/NT reaction, Ti–O-H ⋯ O—and Ti–O-H ⋯ N- attractions are generated between FA and NT. The existence of these attractions increases the lipophilicity and decreases the hydrophilicity of NT.

It is well known that before the FA reaction, the C–Cl bond in PVC is weakly polar, the hydroxyl group in NT is a polar group, and PVC/NT is a weak polar-strong polar unstable composite. The C–Cl bond (weak polarity) in PVC will repulse the OH hydroxyl group (strong polarity) in NT at the interface, which is the main reason for the decline in the thermomechanical properties of the PVC/NT materials in use.

After PVC is added, a relatively stable attraction exists between PVC and FA/NT. XPS analysis shows that C-1 has the best heat resistance and the lowest surface chlorine content of 4.48%, while C-2 and C-3 contain 5.94% and 7.70% chlorine, respectively, which suggests that chlorine is involved in intermolecular bonding in C-1, which leads to a decrease in chlorine content. After doping, the compatibility between PVC and FA/NT is significantly enhanced due to the existence of a stable and strong attraction with Cl atoms.

There are usually two ways to reduce chlorine on the PVC surface: one way is the same as that encountered in this paper, that is, participating in intermolecular bonding and bonding with FA/NT, including attraction (hydrogen bonding), ; the other way involves the removal of hydrogen chloride by thermal degradation, which leads to a reduction of the surface chlorine content of the PVC film.

XPS analysis of S0 and S1 shows that the oxygen content (content%) from large to small follows the order of C-1 (19.38) > S1 (17.8) > S0 (17.01) > C-2 (15.96) > C-3 (14.93). This shows that the surface oxygen content of C-1 is the highest, but the surface oxygen concentrations for S1 and S0 are also high. It can be observed that the oxygen content is not the decisive factor in determining whether a force is generated, and the oxygen content needs to be judged in combination with the chlorine content on the surface.

The chlorine content (content%) from small to large follows the order of C-1 (4.48) < C-2 (5.94) < S0 (6.81) < C-3 (7.70) < S1 (7.74). This shows that the surface chlorine concentration of C-1 is the lowest, which further proves that there is a strong interaction between the intermolecular FA/NT and C–Cl bonds in PVC, resulting in a decrease in the surface chlorine concentration.

## Conclusions

NT modified by FA was synthesized using ultrasonic radiation, and doped PVC polymer films were prepared to reduce the agglomeration of nanoparticles, enhance the compatibility with PVC, and improve the thermal properties. The diffraction patterns measured for the composites show a decrease in the FA/NT peak intensities. As the amount of modified NT is increased from 3 to 5 phr, it becomes incompatible with PVC. The induction period of hydrogen chloride released from modified PVC at 195 °C in a nitrogen atmosphere is much higher than that for the control and blank samples. The minimum temperature required for 1.0% thermal decomposition of the composite film with 1 phr FA/NT-doped PVC is 230.0 °C, which is much higher than the decomposition temperatures of 210.8 °C and 175.1 °C obtained for the control and blank samples, respectively. The peak temperature during the first decomposition stage is 309.5 °C, which moves the decomposition of the PVC into the high-temperature zone. The decomposition rate at this peak temperature is 39.6% and 102.6% lower than that of the control and blank samples, respectively. After adding 1 phr FA/NT, the stability of the PVC composite film is improved due to a lower number of surface chlorine atoms and intermolecular attraction. Compared with the blank sample, the enhanced PVC film suppresses the release of CO_2_, and the vibration peak intensities for ester groups at 1264 cm^−1^, 1736 cm^−1^, 1106 cm^−1^ and 1024 cm^−1^ are attributed to a significant decrease in DOTP, indicating that the FA/NT additive has a strong interaction with DOTP. The strongest vibration peak at 1264 cm^−1^ is reduced by 17.2% compared with that for the blank sample, which shows that the doping of PVC films is beneficial to the recovery of the plasticizer and leads to an improved heat resistance.

## Methods

### Materials

Ordinary commercial PVC (S-65, degree of polymerization, 1000–1100) was obtained from Formosa Plastics Industry (Ningbo) Co., Ltd. (China). The plasticizer dioctyl terephthalate (DOTP) was analytically pure and supplied by Bluesail Group Co., Ltd**.** (Zibo, China). Methyl tin mercaptan (OT) was supplied by Jianhua Dongxu Chemical Co., Ltd. (Quzhou, China). CAS: 57583–35-4, structural formula (I) contains 75% and (II) 25%. Cerium stearate (CeSt3), m.p. 105 °C, cerium content 11.0%, [C_17_H_35_COO^−^]_3_·Ce^+3^ was an industrial qualified product and provided by Zibo Luchuan Rubber & Plastic Co., Ltd. Folic acid, analytically pure, was supplied by Aladdin Biochemical Technology Co., Ltd. (Shanghai, China). Nanotitanium dioxide (NT), anatase type, 20 nm, was supplied by Evonik Degussa AG. The structural formulas for some raw materials are shown below:
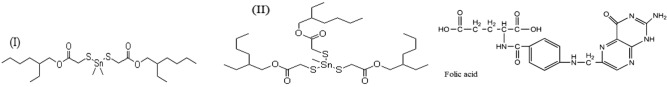


### Characterization

Powder X-ray diffraction (XRD) patterns were recorded with a Rigaku Smart Lab SE diffractometer using a CuKα source (λ = 0.154060 nm) at 40 mA and 40 kV.

Fourier transform infrared spectroscopy was conducted using a Thermo Fisher Scientific Nicolet iS 50 spectrophotometer using KBr pellets (sample/KBr = 1/100), PVC film of 1.0 × 1.0 cm for direct analysis, and a wavelength interval from 4000 to 500 cm^−1^ at a resolution of 4 cm^−1^.

Thermogravimetric (TG) curves were recorded in flowing nitrogen with a heating rate of 10 °C min^−1^ using a Perkin-Elmer Pyris1 thermal analyzer. The temperature range was 25–700 °C, the flow rate of the carrier gas was 100 mL min^−1^, and an alumina crucible was used.

An SX713 conductivity meter was used to automatically display and record the results of the conductivity measurements.

The surface structural changes for the PVC samples were characterized by an X-ray photoelectron spectrometer (XPS), model: Thermo Scientific ESCALAB 250Xi. An incident X-ray beam from an Al target (with an accelerating potential of 10 keV and a current of 4 mA) was focused onto the surface of the sample with an electron take-off angle of 90°.

TG-FTIR: The pyrolysis behavior of PVC was analyzed by using a thermogravimetric analyzer (TGA, Perkin Elmer 8000)-Fourier transform infrared spectrometer (FTIR, Frontier). To reduce the heat transfer limitations, approximately 5.0 ± 0.1 mg of sample was added to a platinum crucible for TGA and heated from room temperature to 700 °C at a heating rate of 10 °C min^−1^ under high purity N_2_ (99.999%) at a flow rate of 100 mL min^−1^. The gas emitted during pyrolysis was transported from the TGA to the FTIR spectrometer through a capillary bundle while the temperature was maintained at 200 °C. FTIR data were recorded in the wavenumber range of 4000–500 cm^−1^ at a resolution of 4 cm^−1^.

### Determination of the hydrogen chloride concentration

The concentration of the aqueous solution of hydrogen chloride released during PVC pyrolysis was determined by conductivity conversion. The corresponding relationship between the concentration of [HCl] in the absorbing solution and the conductivity value, that is, the calculated [HCl], can be expressed as (1)^37^:

1 $$\left[ {{\text{HCl}}} \right]_{{\text{t}}} = \left[ {0.{5}0{1}\;{\text{V}}_{{\text{t}}} + 0.0{1}} \right] \, \times {1}0^{{ - {5}}} \,{\text{mol}}\;{\text{L}}^{{ - {1}}}$$

where [HCl]_t_ is the molar concentration of the HCl solution at time t, V_t_ is the difference between the conductivity of the HCl solution at time t and the conductivity of the deionized water, and 0.01 is a correction value that takes into account the presence of CO_3_^2−^ ions dissolved in water^37^; additional information for the conductivity measurement is supplied in the Supporting Information.

### Synthesis of FA/NT

The mass fraction of FA in the FA/NT composite was 5%, and the required amounts of FA powder and NT were each weighed, dissolved in deionized water at 25 °C, and ultrasonicated for 15 min. Each solution was then poured into the same container, mixed, and stirred. Then, ultrasonication was conducted for 30 min at 25 °C, evaporative dehydration was performed, and the samples dried at 105 °C in vacuum to obtain the FA/NT particles.

### Preparation of the PVC film

The plasticizer (DOTP), thermal stabilizer (OT), costabilizer (CeSt_3_), and reinforcing agent (FA (5%)/NT) were weighed in proportion, combined at 80 °C and ultrasonicated for 30 min to fully mix the materials and form a uniform viscous liquid. The viscous liquid was added to the PVC resin and mixed at 170–175 °C at a speed of 40 r•min^−1^. The mixing torque was first rapidly increased and then decreased, and after mixing for 5 min, the PVC discharge was removed and compressed to a thickness of 1.0 mm using a flat vulcanizing machine at 100 °C to obtain a PVC sheet, as shown in Table 6.

**Table 6:** Composition of the PVC film.

Sample	PVC resin	DOTP	OT	CeSt_3_	FA/NT
	/phrs				
S0	100	50			
S1	100	50	1.0		
C-1	100	50	0.5	5.0	1.0
C-2	100	50	0.5	5.0	3.0
C-3	100	50	0.5	5.0	5.0

The FA content in FA/NT was 5 wt%, S0 is the blank sample, and S1 is the control sample.

### Conductivity measurement

The conductivity was measured in accordance with the ENIS0182-3:2000 standard. A PVC sample with a size of 2.0 × 2.0 × 1.0 mm, weighing 2.2000 g, was added to a test tube. The test tube was placed in a silicon oil bath, and N2 carrier gas flowed at a rate of 100 ml/min. Sixty milliliters of deionized water was placed in a container. A conductivity meter (model SX-731) was used to measure the conductivity of the deionized water. The temperature of the PVC sample in the test tube was set to 195 °C, and nitrogen was used to purge the HCl gas that escaped after decomposition. Hydrogen chloride was dissolved in deionized water; the conductivity change in the deionized water during the entire heating process was automatically recorded by a conductivity meter, and the isothermal degradation test ended when the conductivity increased to 50 μS·cm^−1^.

### Characterization of FA/NT

#### FT-IR

**Figure 11:**
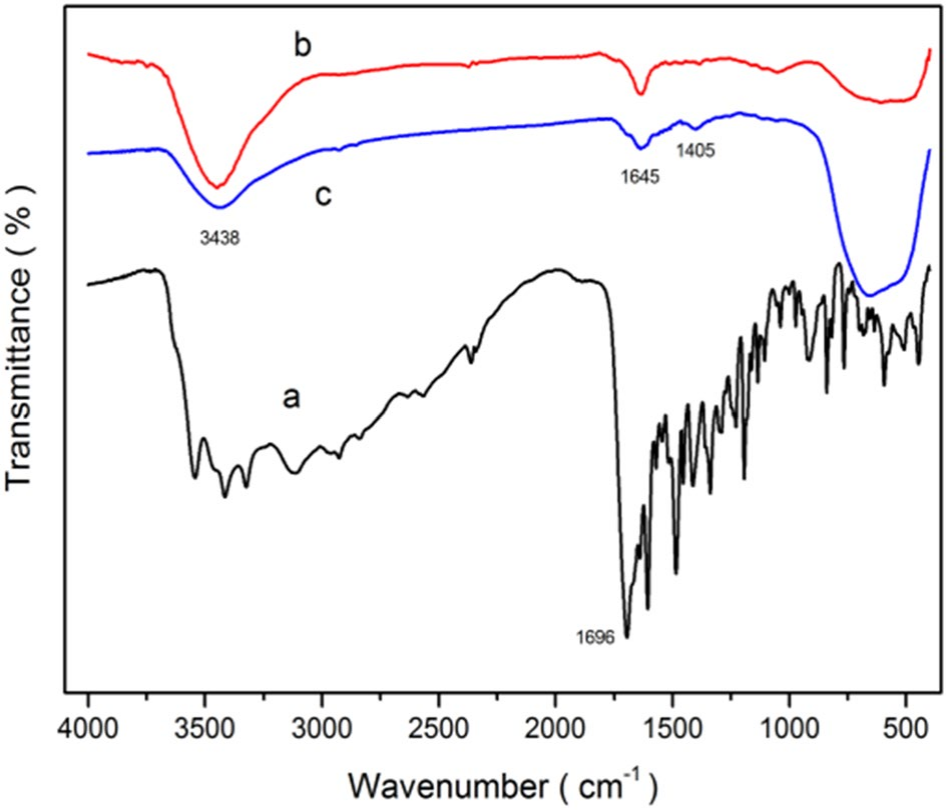
The FT-IR spectra for (**a**) FA, (**b**) NT and (**c**) FA/NT.

Figure 11 shows the infrared spectra for folic acid (FA), nanotitanium dioxide (NT), and folic acid-modified nanotitanium dioxide (Fa/NT). a, b and c represent FA, NT and FA/NT particles, respectively. The vibration peaks a, b and c at 3438 cm^−1^ and 1645 cm^−1^ are related to hydroxyl groups, wherein the intensity of c is significantly lower than that of b, indicating that the vibrations of the hydroxyl groups are reduced after the reaction. The broad peaks observed in the wavenumber range of 450–800 cm^−1^ are attributed to Ti–O-Ti stretching vibrations. By comparing the NT spectra before and after modification, it can be found that there are differences near 1404 cm^−1^. In the range of 400–1000 cm^−1^, the vibration peak intensity increases, indicating that interactions occur between FA and NT^44,45^.

### XRD

**Figure 12:**
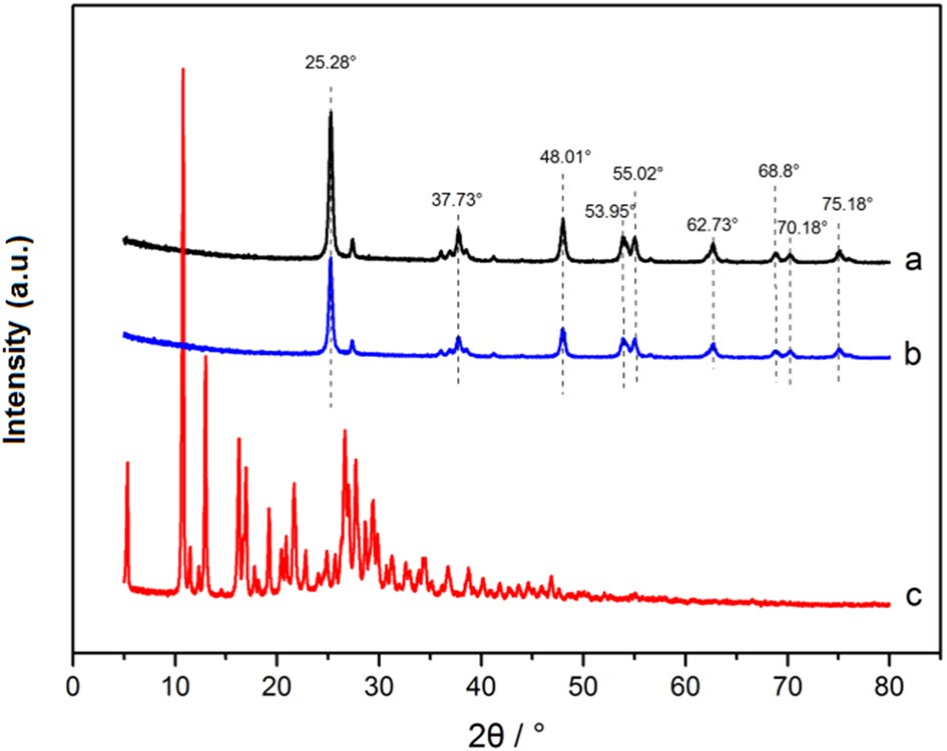
XRD patterns for (**a**) NT, (**b**) FA (5%)-NT and (**c**) FA.

Figure 12 shows the XRD diffraction patterns for NT, FA (5%)-NT and FA. The characteristic peaks for NT anatase appear at 2θ = 25.3°, 37.8° and 48.0°; the characteristic peaks for the rutile type appear at 2θ = 27.4°, 36.1° and 54.3°. The spectrum shows that a rutile-type NT peak appears near 27.4°, which indicates the presence of an anatase-type NT that contains a small amount of rutile NT. The characteristic peaks for NT observed at 25.3°, 37.7°, 48.0°, 53.9°, 55.1°, 62.7°, 68.8°, 70.2° and 75.1° are attributed to the (101), (004), (200), (105), (211), (204), (116), (220) and (215) NT diffraction peaks, respectively^6,45^. The diffraction peaks observed for NT modified with FA almost coincide with the diffraction peak observed for NT before modification. FA/NT basically retain the characteristic NT peak, but the peak intensity is lower than that of NT. These diffraction peaks correspond to the peaks obtained for anatase-type NT, so the prepared FA/NT is still anatase-type NT, and the addition of FA does not obviously change its crystalline properties. For a 2θ of 10.79°, the FA powder shows the largest diffraction peak. The addition of FA leads to a FA/NT peak intensity that is lower than that of NT. The average crystallite size of NT before and after modification was calculated according to the Debye–Scherrer formula (2)^46^:

2 $${\text{d}} = {\text{k}}\lambda /\left( {\beta {\text{cos}}\theta } \right)$$

where *d* is the microcrystalline size, *k* is 0.89 (CuK), *λ* is the wavelength of the X-ray (*λ* = 0.15418 nm), *θ* is the Bragg diffraction angle, and *β* is the full width at half maximum (FWHM). For a 2θ of 25.28°, the average microcrystalline sizes before and after modification are calculated from the diffraction peaks to be 21.08 nm and 19.78 nm, respectively. The results show that the average grain size of the FA/NT particles is decreased by 1.3 nm.

#### TG

**Figure 13:**
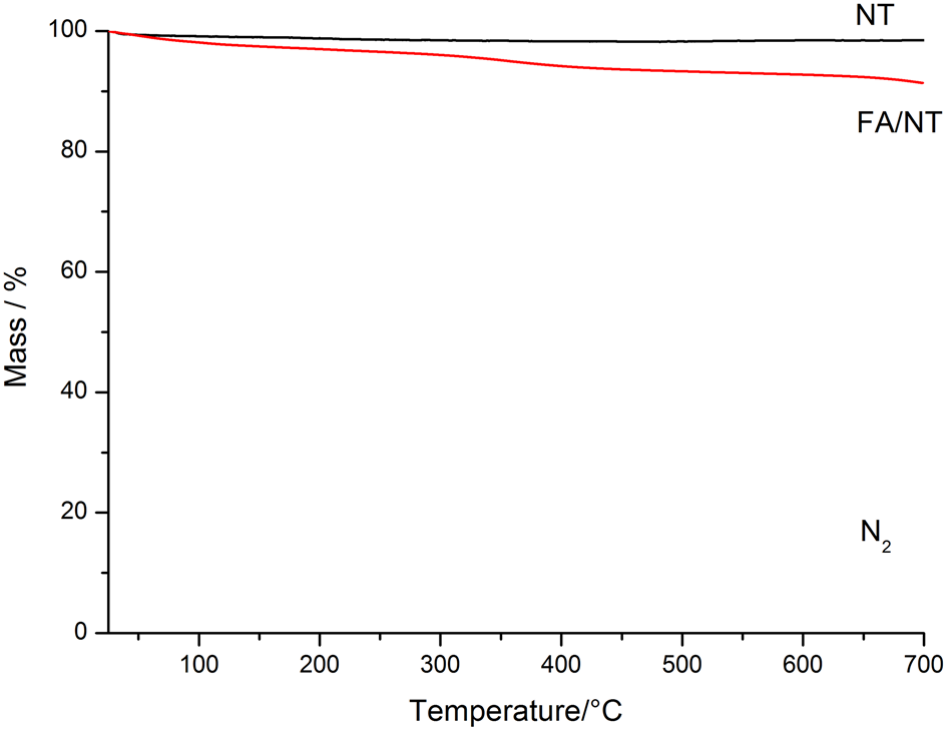
TG curves for NT and FA/NT.

Figure 13 shows the TG curves for NT and FA/NT. The heating rate was 10 °C min^−1^ in a nitrogen atmosphere, and as shown in the figure, NT shows a small mass loss during heating. At 700 °C, the residual mass is 98.46%, and the mass loss due to loss of moisture and volatile substances is 1.54%; during the heating process, the residual mass of FA/NT at 700 °C is 91.36%, and the mass loss is 8.64%.

#### XPS

**Figure 14:**
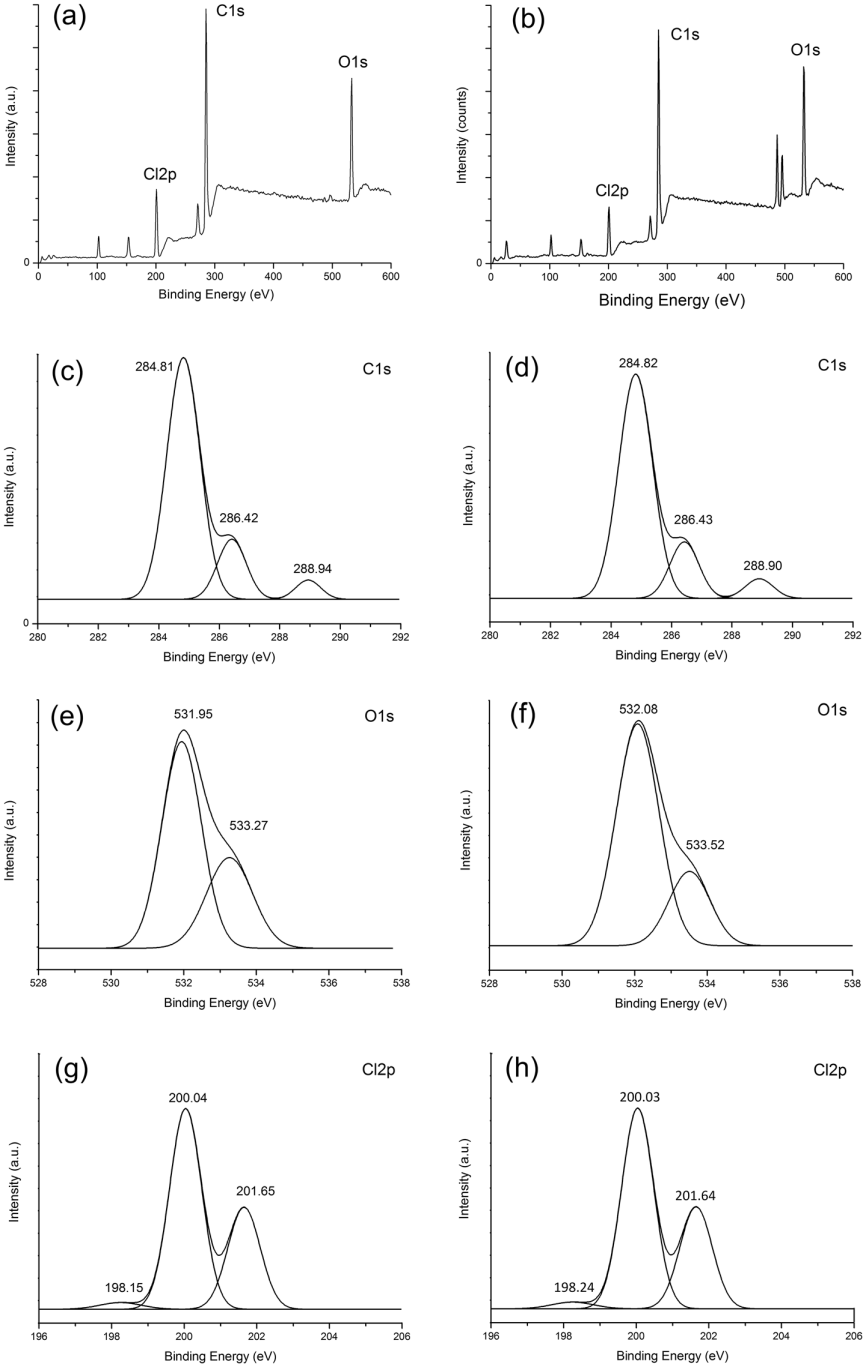
The XPS spectra for PVC films (Note: The wide spectrum of film (**a**) S0 and (**b**) S1; C1s spectra of film (**c**) S0 and (**d**) S1; O1s spectra of film (**e**) S0 and (**f**) S1; Cl2p spectra of film (**g**) S0 and (**h**) S1).

Figure 14 shows the XPS spectra for films S0 and S1. As can be observed from the figure, the C 1s, O 1s and Cl 2p spectra for S0 and S1 are similar with little difference. XPS spectra obtained from high-resolution scans of the C 1s region for (a) S0 and (b) S1 are also shown. From the C 1s spectra for the S0, C-2 and C-3 films, the three components 1, 2 and 3 in the C ls spectra correspond exactly to three types of carbon bonds: C–C (284.81 eV), C–O and C-N (286.42 eV) and amide C=O (288.94 eV), respectively.

As displayed in Fig. 14e, two different types of oxygen components, O=C (531.95 eV) and O–C (533.27 eV), are present in the O 1 s spectrum of the S0 film. From the spectrum obtained from high-resolution scans of the O 1s region of S1 film shown in Fig. 14f, two different types of oxygen components are observed to show no significant change. The high-resolution C l2p spectrum for the S0 film shows three different chlorine components at 198.15 eV, 200.04 eV and 201.65 eV shown in Fig. 14g,h. From the high-resolution Cl 2p spectrum of the S1 film, three different chlorine components are observed to show no significant change.
